# Unveiling Langerhans cell histiocytosis presenting as hidradenitis suppurativa: A case report and systematic review

**DOI:** 10.1016/j.ijscr.2024.110758

**Published:** 2024-12-26

**Authors:** Ibrahim Mohammadzadeh, Narges Bazgir, Behnaz Niroomand, Ghaem Khodabakhsh, Nader Akbari, Seyed Ali Mousavinejad

**Affiliations:** a Skull Base Research Center, Loghman-Hakim Hospital, Shahid Beheshti University of Medical Sciences, Tehran, Iran; b Hearing Disorders Research Center, Loghman Hakim Hospital, Shahid Beheshti university of Medical Sciences, Iran

**Keywords:** Langerhans-cell histiocytosis, Suppurative hidradenitis, Rare dermatological disorders, Pituitary lesions, Neurosurgery

## Abstract

**Introduction and importance:**

Langerhans cell histiocytosis (LCH) is a rare disorder characterized by the proliferation of abnormal Langerhans cells, often presenting with symptoms that mimic common dermatological conditions such as hidradenitis suppurativa (HS). Accurate diagnosis is essential because LCH can affect multiple organ systems and necessitates distinct therapeutic approaches.

**Case presentation:**

We report a rare case of a 39-year-old male with a 7-year history of diabetes insipidus (DI), who presented with polyuria, polydipsia, and enlarging purulent lesions in the axilla and groin. MRI revealed a pituitary lesion, and subsequent histopathological examination confirmed LCH. The patient underwent surgical interventions to address recurring cerebrospinal fluid (CSF) leaks and manage the underlying LCH.

**Clinical discussion:**

Histopathology confirmed LCH with positive markers for CD1a and S-100 proteins. Post-surgery, the patient's symptoms, including polyuria and polydipsia, resolved without complications. There was no recurrence of CSF leakage or other LCH-related symptoms during follow-up. This case illustrates the diagnostic challenges of LCH when it mimics common conditions such as HS and underscores the importance of a multidisciplinary approach, particularly when standard treatments are ineffective.

**Conclusion:**

Surgical intervention was pivotal in the resolution of symptoms, highlighting the necessity for timely and accurate diagnosis to improve outcomes in multifocal LCH cases.

## Highlights

• Langerhans cell histiocytosis (LCH) mimicked hidradenitis suppurativa (HS), causing an initial misdiagnosis.
• Persistent inflammatory skin conditions should consider LCH in differential diagnoses.
• Surgical intervention resolved symptoms effectively in this rare LCH case.

## 1 Introduction

Langerhans cell histiocytosis (LCH) involves the abnormal growth of Langerhans immune cells and is frequently misdiagnosed. Proper recognition is vital to manage the converging symptoms and to provide the correct treatment [1]. The condition can range from skin rashes to bone lesions and, in severe cases, may affect major organs like the pituitary gland and lungs. A case study highlights the complex relationship between skin symptoms and a rare pituitary mass. LCH can invade the pituitary region in 5 %–50 % of cases, potentially causing central diabetes insipidus and pituitary deficiencies [2,3]. The commonly observed deficiencies in these cases include a high prevalence of growth hormone (GH) deficiency (53–67 %), low thyroid-stimulating hormone (TSH) levels (3.9 %), and gonadotropin deficiency (53–58 %) [[4], [5], [6]]. When erosive lesions, characterized by open sores or wounds, appear in the axilla and inguinal region, it often raises suspicion of hidradenitis suppurativa (HS) [7]. In this report, we present a rare case of solitary pituitary stalk LCH that was initially misdiagnosed as HS, highlighting the diagnostic complexities when a common dermatological condition masks an underlying systemic disorder. Additionally, through a systematic review, we aim to explore the intersection of these two conditions, offering new insights into the clinical management and diagnostic approaches for patients presenting with similar symptoms. This case and review provide a fresh perspective on the potential link between chronic inflammatory skin conditions and LCH, expanding the current understanding of its diverse manifestations. This case report has been reported in line with the SCARE criteria [8].

## 2 Case presentation

**Fig. 1:**
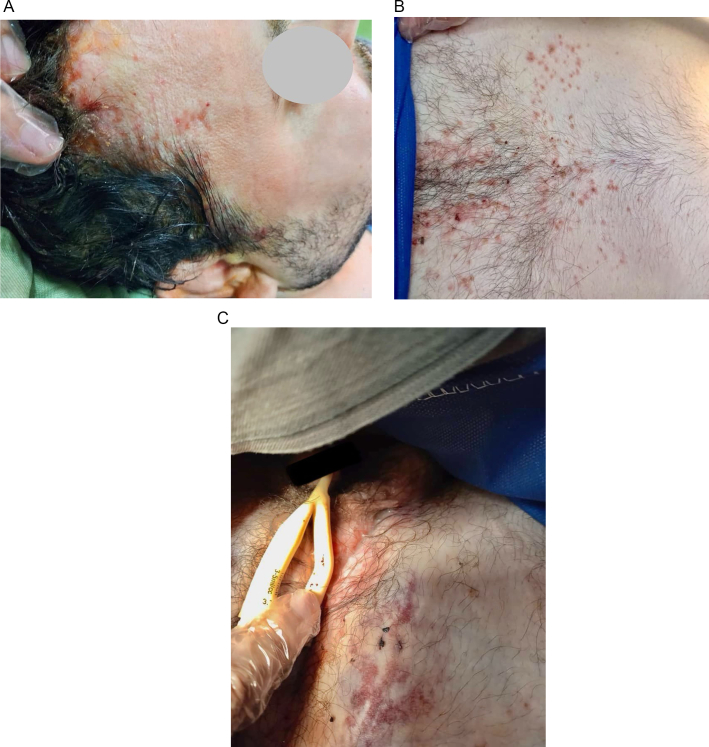
A. Pre-operative clinical photograph showing purulent lesions and inflammatory skin changes on the patient's scalp. B. Pre-operative clinical photograph showing inflammatory skin lesions and nodules in the patient's lumbar region. C. Pre-operative clinical photograph of purulent lesions and skin changes in the patient's groin region.

A 39-year-old male patient, diagnosed with diabetes insipidus (DI) for the past 7 years, presented with complaints of polyuria, polydipsia, and progressive enlargement of purulent hidradenitis in the scalp, lumbar and groin regions (Fig. 1 A, B, and C). His habitual and family histories were insignificant. The patient's clinical chronicle revealed a surgical intervention for a cerebral adenoma conducted at our institute, on the 31st of October 2023, which was followed by postoperative complications related to mesh removal, leading to symptoms of CSF leakage. Subsequent surgery on 25th November of 2023 involved the use of a nasoseptal flap for leak repair, with a recurrence of symptoms due to the patient's non-compliance with postoperative recommendations. Physical examinations revealed symptoms such as decreased libido, weight gain, moon face, polyuria, gynecomastia, and hypothyroidism. Besides, his neurological examinations were normal. Although the patient presented with regular oculomotor activity, there was an occurrence of maculopapular lesions. Exanthema distributed across the cranial, facial, cervical, lumbosacral, axillary, and inguinal areas.

**Fig. 2:**
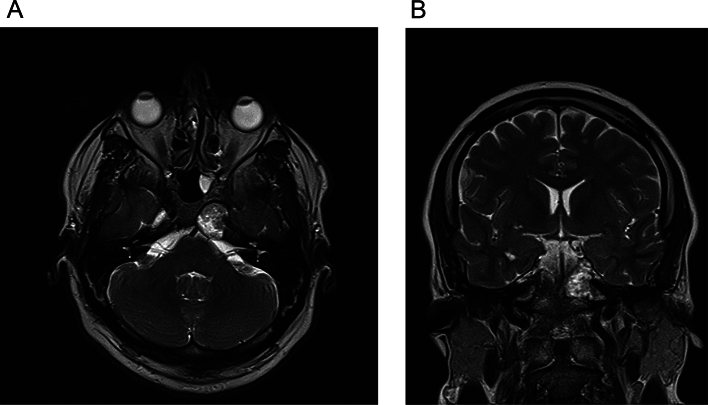
A. Pre-operative transverse MRI image showing an 18x17x12 mm lesion in the hypothalamic region and proximal pituitary stalk, with mild surrounding tissue swelling and displacement of adjacent structures. B. Pre-operative coronal MRI image illustrating the lesion involving the hypothalamic-pituitary region with associated mass effect on nearby structures.

**Fig. 3:**
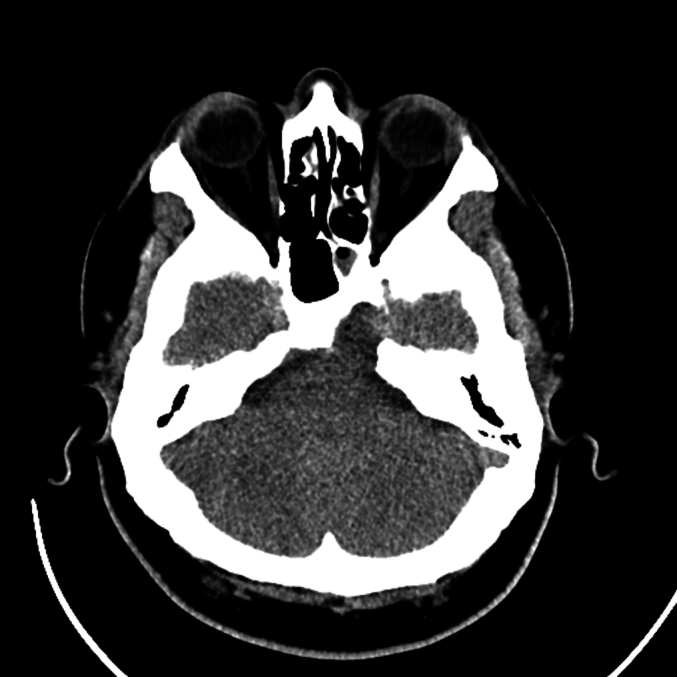
Pre-operative axial CT scan revealing a hypodense lesion in the hypothalamic region and proximal pituitary stalk, with no significant involvement of surrounding bone structures.

A thorough laboratory investigation was performed (Table 1). The patient's imaging (magnetic resonance imaging (MRI), and the Computed Tomography (CT) revealed an 18*17*12 mm lesion in the hypothalamic region and the proximal pituitary stalk (Fig. 2, Fig. 3). Systemic workups showed no other involvements.

**Table 1:** Laboratory test results of the patient, comparing key blood parameters with normal ranges.

Lab test	Result	Normal ranges
RBC^a^	5.1*10^6	4.35-5.65 *10^6
WBC^a^	8200	4500-11,000
PLT^a^	356,000	150,000-450,000
Hb^a^	14.2	12-16
Albumin	4.4	3.4–5.4
Bilirubin	0.8	0.1–1.2
Aspartate aminotransferase	23	8–33
Alanine transaminase	22	19–25
Creatinine	0.9	0.7–1.3
Na	140	135–145
K	4.8	3.5–5.2
PT^a^	12	11-13.5
PTT^a^	25	25-35
ESR^a^	42	<15

a Abbreviation: Red blood cell count: RBC, White blood cell count: WBC, Platelet count: PLT, Hemoglobin: Hb, Prothrombin time: PT, Partial thromboplastin time: PTT, Erythrocyte sedimentation rate: ESR.

**Fig. 4:**
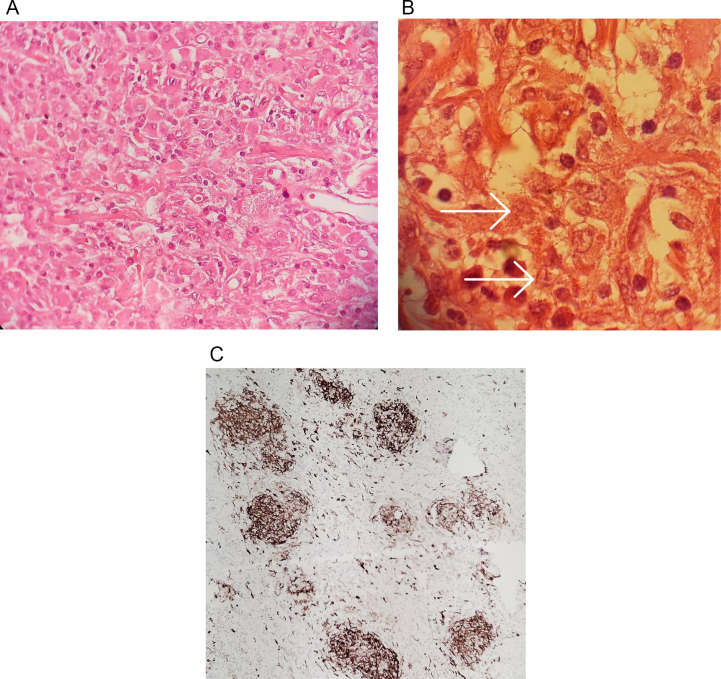
A. Langerhans cells admixed with some lymphocyte and eosinophils, ×400, H&E stained. B. Langerhans cells with horse shoe and bilobed nuclei (arrows)and scattered lymphocytes and eosinophils, ×1000, H&E stain. C. CD1a positive Langerhans cells, ×400.

Histopathologic examination revealed a neoplasm characterized by sheets of ovoid cells with abundant eosinophilic cytoplasm and indented reniform nuclei with few mitotic figures, no necrosis and admixture of other inflammatory cells including lymphocytes, eosinophils, neutrophils, mast cells and neoplastic cells are positive for CD1a, S100, CD68 with low proliferative index and negative for GFAP, Synaptophysin and consistent with the diagnosis of LCH (Fig. 4 A, B and C).

## 3 Surgical procedure

**Fig. 5:**
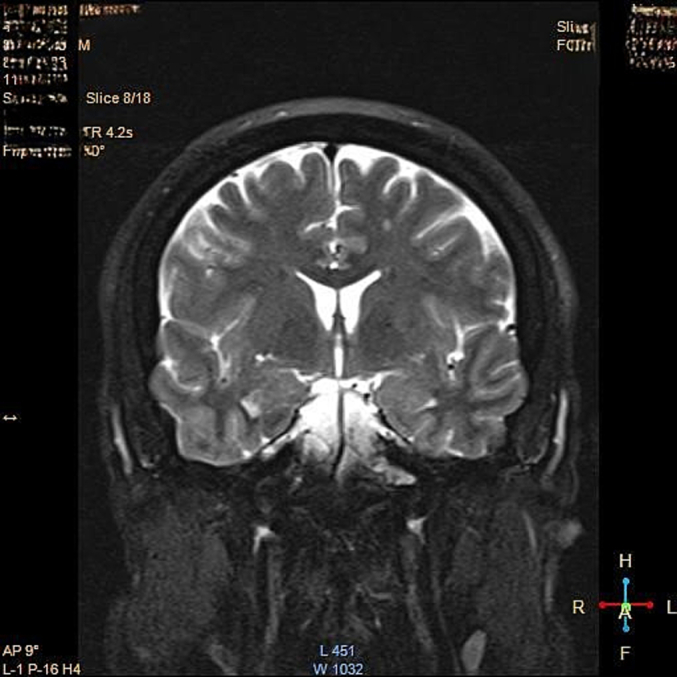
Post-operative coronal MRI image showing resolution of the lesion in the hypothalamic-pituitary region after surgical intervention, with no visible residual mass or significant postoperative complications.

**Fig. 6:**
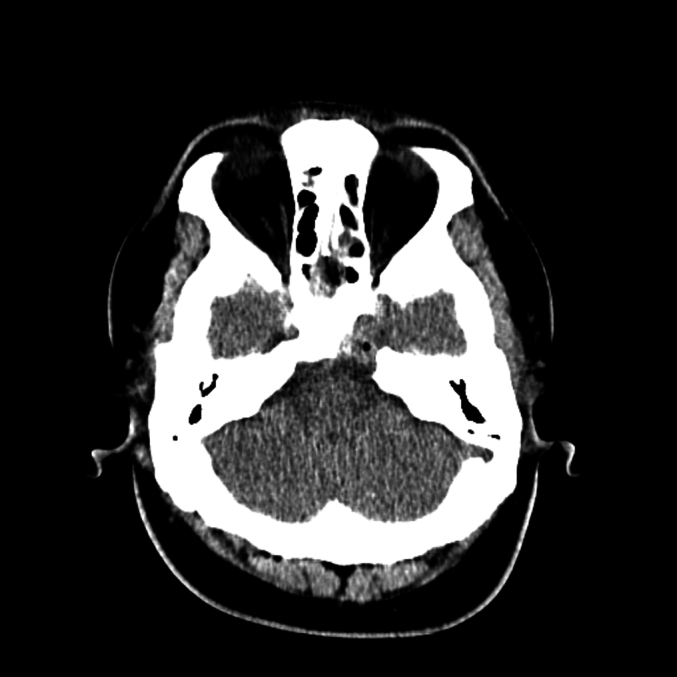
Post-operative axial CT scan confirming the absence of any remaining mass in the hypothalamic region.

Initially, the right nasal cavity was meticulously examined using an endoscope equipped with a zero-degree lens, addressing the right septal deviation. Subsequently, the resection of the lower half of the right middle turbinate was undertaken, ensuring effective hemostasis. Identification of the medial opening of the sphenoid sinus, situated adjacent to the upper turbinate of the nose, revealed a broad sphenoid sinus aperture. Sequentially, both posterior and anterior ethmoidectomies were executed. A posterior nasal septectomy was performed, rendering both nasal passages accessible, thus adopting a binostril surgical approach. Thorough drilling of the sphenoid face was carried out to ascertain the presence of internal carotid components, the Sella region, and the intrasellar recess. Extensive drilling of the Sella area facilitated precise determination of the distance to the tumour. With utmost care, the dura was incised, allowing for complete drainage of the tumour. Strategically, fat and fascial materials were strategically placed in the Sella region to manage CSF leak. Hemostasis was meticulously achieved, culminating in the placement of a nasal mesh. In the second phase of the procedure, the repair of the CSF leak was followed by the removal of the nasal mesh, which had been previously undertaken at another facility without coordination with the primary surgeon. Subsequently, the patient experienced a clear runny nose within two days, prompting readiness for the operation. The initial steps involved draping the operation area and nose, followed by multiple washes with diluted gentamicin and sterile serum. Subsequent actions included minor drilling around the cell and fat area, with the smoothing of wound edges to facilitate repair. Harvesting fat from the abdomen and concurrent removal of fascia enabled a three-layered CSF leak repair using fat, fascia, and gel foam. The final step encompassed the placement of nasal mesh, concluding the operation. Moving on to the third stage, following a ten-day observation period, the patient exhibited recurring symptoms of CSF leak, necessitating another operation. A thorough examination of the Abenda Sela area revealed no evident leak. Subsequently, the patient's position was altered to Trendelenburg, respiratory volume increased, and the Valsalva manoeuvre was performed to induce increased intracerebral pressure, ultimately revealing a suspicious area of cerebrovascular fluid. Despite the absence of a clear leak at a specific point, the area was slowly filled with CSF. To address this, slight drilling around the cell and its surroundings was conducted to cover the wound edges. Additionally, a nasoseptal flap, derived from the opposite side of the nose (left side) based on the posterior septal artery, was removed and orthotopically placed in the nasopharynx. Gel foam and nasal mesh were then employed to secure the flap in position, completing the repair process. Post-operative MRI and CT images can be observed in Fig. 5, Fig. 6.

During the follow-up period, the patient was stable with no headache, or cognitive disorders. His symptoms were resolved and he continued normal daily activity.

## 4 Literature review

### 4.1 Method

The purpose of this article was to systematically review the cases that suffered from LCH but manifested HS symptoms. The study was conducted following The Preferred Reporting Items for Systematic Reviews and Meta-Analyses (PRISMA) guidelines [9].

### 4.2 Eligibility criteria

All English studies that reported cases of LCH masquerading as HS were included, while non-English articles, reviews, book chapters, conference abstracts, and articles not related to LCH mimicking HS were excluded.

### 4.3 Search strategy

A systematic search using relevant keywords, including “Langerhans-cell histiocytosis,” “Pituitary lesion,” “Skin,” “Histiocytosis,” and “Hidradenitis suppurativa,” was conducted in the PubMed, Scopus, Embase, and Google Scholar databases on October 16, 2024. After gathering articles from each database, we imported the records into an Excel spreadsheet. We then conducted a thorough review to identify and remove any duplicate articles. Next, we carefully screened the studies based on specific eligibility criteria, using both the title and abstract to assess their relevance. Subsequently, we selected articles that were deemed suitable for a comprehensive full-text assessment. Finally, we used the articles that met the inclusion criteria for data extraction and analysis.

## 5 Results

### 5.1 Study characteristics

**Fig. 7:**
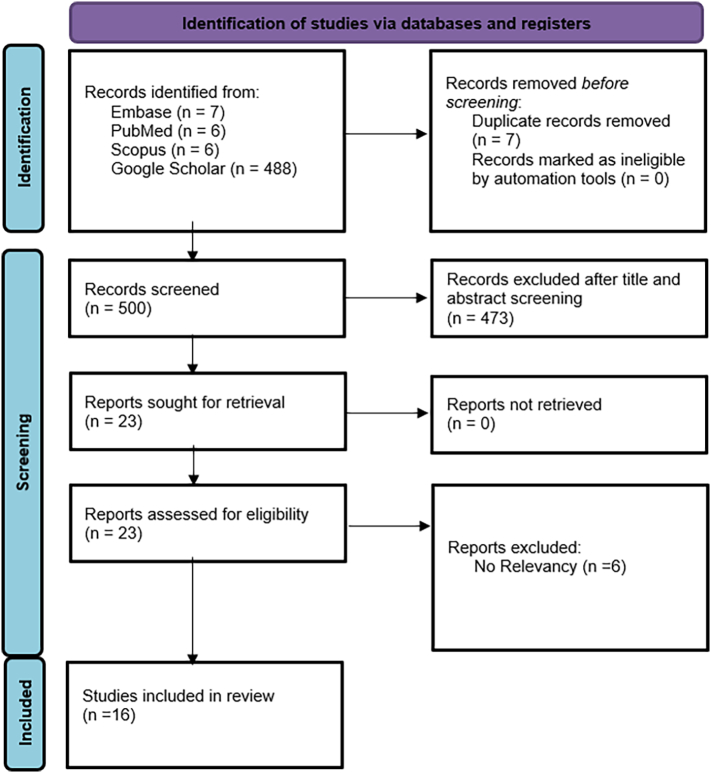
PRISMA flowchart of the study selection process.

After conducting comprehensive research in PubMed, Scopus, Embase, and Google Scholar, by using the initially 507 articles were identified. Seven duplicated studies were identified. Leaving 500 articles for screening. After title and abstract screening 473 articles were excluded. Twenty-seven studies remained for full-text screening. Finally, sixteen studies including 16 cases with LCH masquerading HS were included in the review (Fig. 7).

This review includes several case reports of LCH in male and female patients, with a mean age of 37.47 years, most of whom were male (male-to-female ratio: 1.83). The majority were diagnosed with multisystem involvement, presenting symptoms such as skin lesions, painful ulcers, diabetes insipidus, and recurrent pneumothorax. Imaging revealed widespread cystic lesions in the lungs and pituitary abnormalities like stalk thickening. Treatments included chemotherapy agents like cytarabine and vinblastine, along with systemic corticosteroids.

### 5.2 Quality synthesis

The results of conducted search are demonstrated in Table 2.

**Table 2:** Summary of selected cases of LCH mimicking Hidradenitis Suppurativa, showing patient demographics, clinical manifestations, imaging findings, pathology results, and treatment approaches.

First author	Gender	Age	Manifestations	Imaging	Pathology	Treatment
Ferrara G et al. [7]	M^a^	32	Several papules on the scalp, face, and trunk, with nodular lesions in the axillae, indicating LCH with multisystem involvement	N/M	A large population of diffuse histiocytoid cells in the superficial, middle, and deep dermis, tested positive for anti-CD1A and anti-S100.	N/M^a^
Kalen E et al. [13]	M	32	Diabetes insipidus, spontaneous pneumothoraces, pleural effusion, and painful exudative ulcers in axillary and inguinal folds, followed by inflamed cystic lesions with purulent drainage, indicating LCH with multisystem involvement	N/M	Langerhans cells with large nuclei, abundant pink cytoplasm, and numerous eosinophils. Immunohistochemistry confirmed CD1a, CD4, S-100 (strong, diffuse), and focal CD68 reactivity.	Cytarabine, and clindamycin 1 % and fluocinonide 0.05 % for skin lesions
Chertoff et al. [14]	M	32	Diabetes insipidus, worsening dyspnea and chest tightness Diagnosed with multifocal LCH	In chest radiography diffuse reticulation and cystic lesions of both lungs In CT^a^ diffused bilateral irregular cyst	Langerhans cell histiocytosis	cytarabine
Yasuda M et al. [15]	M	42	Painful nodules and ulcers, recurrent draining sinus on the axillary and perianal area Diagnosed with multifocal LCH	Soft tissue from the right neck to the back, thyroid, perianal region, and adductor magnus muscle confirmed by PET^a^/CT tomography	The tissue showed dense infiltration of histiocytic mononuclear and multinucleated giant cells from the dermis to subcutaneous tissue. Histiocytic cells expressed CD1a and S-100 proteins	Chemotherapy
Lian et al. [16]	M	34	Bilateral axillary ulcers, chest congestion, weakness, diabetes insipidus, polyuria, polydipsia, sexual dysfunction, moon face, and exophthalmos, indicating LCH with multisystem involvement	Pituitary MRI showed cerebrospinal fluid in most of the pituitary fossa, reduced volume, and thickened infundibulum with uneven Sella changes. ECT bone scan revealed irregular radioactive concentration in the sternum, ilium, and hip joint, indicating active bone metabolism. CT found an 8 mm T2 high signal nodule in the right orbital wall. Breast CT revealed higher lung field penetrance, uneven cystic shadows, and thickened interlobular septum. Abdominal ultrasound showed liver enlargement	Langerhans cells diffusely infiltrated in the dermis and the tumour cells were positive for CD1a and S-100 expression.	Cytoxan, Adriamycin, vincristine, prednisone and etoposide
Mahzoon et al. [17]	M	20	Axillary and inguinal ulcer. Diffuse infiltrations and restrictive lung disease. LCH with multisystem involvement	N/M	Langerhans cell histiocytosis	N/M
Ang C et al. [18]	F	23	Recurrent pneumothorax, chronic vulval and right axillary ulcers, hypernatremia, hyperosmolarity with low urine osmolarity, indicating LCH with multisystem involvement	Slight thickening of the pituitary stalk with loss of posterior pituitary hyperintensity; multiple thin-walled lung cysts, left pneumothorax, and subcutaneous emphysema	Infiltration of large mononuclear cells with vesicular, reniform and grooved nucleus in the dermis, together with eosinophils and lymphocytes; immunohistochemical staining for CD1a antigen	Systemic chemotherapy
Goodfello et al. [19]	M	31	Bilateral sensorineural hearing loss, rashes on the face, painful axillary ulcers, polyuria, polydipsia, exertional dyspnea, and destructive bone lesions. LCH with multisystem involvement	Chest X-ray showed mottled reticular shadowing and small nodules throughout the perihilar region and the base of the lung	Langerhans' cell granules within infiltrating histiocytes	Desmopressin, prednisolone, nitrogen mustard
Hoang et al. [20]	F	46	Axillary and genital purulent ulcers	N/M	Langerhans' cell granules	N/M
Modi D et al. [21]	F	65	Skin ulceration in the left axilla, groin, intergluteal folds, and ano-perineal region, with palpable axillary lymph nodes	X-rays of the chest, skull, skeletal survey, and total body bone scan were all normal	The biopsy showed dermal inflammation with prominent eosinophils and abnormal histiocytes with large irregular nuclei. Many histiocytes stained positive for S100	orthovoltage radiation
Poelman et al. [22]	M	71	Inguinal, perianal, and axillary ulcers, jaw granulomas, hypoparathyroidism, weight loss, night sweats, polydipsia, polyuria, no bone involvement	N/M	infiltration of cells in the superficial to mid-dermis with reniform nuclei and positive immunohistochemical staining for CD1a and S-100	vinblastine
Chiu et al. [23]	M	28	Chronic bilateral axillae ulceration with discharge, acneform eruption covered with yellowish scale.	CT and systemic workup showed no abnormality.	Mononuclear cells reinform nuclei stained positive for CD1 and S-100	Prednisolone and Thalidomide
Yousif et al. [24]	F	34	Persistent anal fissure, painful nodules and plaques in bilateral groin, genitalia, and upper gluteal cleft	MRI showed a pituitary mass.	Histiocytic cells with enlarged irregular nuclei, positive for CD4, CD68, CD1a, S-100	cytarabine
Ataya A et al. [25]	M	30	Cystic and nodular lungs, bilateral recurrent pneumothoraces, diabetes insipidus, multiple pink and violaceous ulcerated tender nodules in both axillae	Chest CT diffuse cystic lung	Histiocytes with reniform nuclei positive for S-100, CD1a	cytarabine
Gunarath et al. [26]	F	53	Recurrent erythematous papules, ulcerative nodules, crusted plaques of scalp, diabetes insipidus	Imaging showed no lung, liver, and bone marrow involvement.	N/M	chemotherapy
Shukla et al. [27]	F	25	Swelling of neck loss of appetite, weight loss, recurrent furuncle	X-ray and ultrasound showed no issues, but a PET scan revealed active disease in soft tissues, bones, muscles, lymph nodes, and lungs	Dense infiltrate of Langerhans cell positive for CD1a	Prednisolone and vinblastine
Mohammadzadeh et al.	M #	39	Polyuria, polydipsia, purulent hidradenitis in axilla and groin, gynecomastia, decreased libido, moon face, weight gain, hypoparathyroidism, and maculopapular rashes.	A mass localized in the hypothalamic region and in close proximity to the proximal pituitary stalk	Ovoid cells with eosinophilic cytoplasm, indented reniform nuclei, positive for CD1a, S100, CD68 (low proliferation), and negative for GFAP and Synaptophysin, consistent with Langerhans-cell histiocytosis	Surgical intervention

a Abbreviation: positron emission tomography: PET, computed tomography: CT, Male: M, Female: F, Not mentioned: N/M.

As demonstrated in Table 2, 11 out of 16 patients were male. Almost all patients suffered from painful skin ulceration, nodules, and other dermatological manifestations. The most frequently affected site was the axilla, followed by the inguinal and groin regions. Other common clinical manifestations included polydipsia, polyuria, diabetes insipidus, and lung involvement. Seven patients had polydipsia, polyuria, and DI like our case. The diagnosis of LCH was confirmed by pathological investigations. The histopathological examinations showed histiocytes that were positive for CD1a and S-100 (Fig. 4 A, B, and C).

Since many of the patients suffered from multifocal and multisystem LCH, the majority of them received chemotherapy. Only one case received radiotherapy. In the presented case, the LCH was resolved by surgical intervention.

## 6 Discussion

This report presents a rare case of LCH which was initially misdiagnosed as HS due to similar symptoms. It underscores the need to consider LCH when treating persistent HS. The case study of a recurrence of CSF leak after multiple surgeries demonstrates the critical need for careful postoperative care and patient compliance with follow-up recommendations in LCH treatment.

Histiocytosis is a group of rare disorders characterized by the overproduction of white blood cells known as histiocytes. These disorders are classified as inflammatory myeloid neoplasms, originating from common myeloid progenitor cells. Histiocytosis encompasses a wide spectrum of diseases affecting various cells of the mononuclear phagocytic system. These disorders are further categorized into dendritic cell (DC) disorders, macrophage-related disorders, and malignant histiocytic disorders [10].

LCH, previously known as histiocytosis X, is a rare haematological disease with diverse manifestations in adults. In adults, LCH is grouped into four subtypes: unifocal, single-system pulmonary, single-system multifocal, and multisystem diseases [1].

The incidence of LCH is about 1–2 cases in a million adults [11,12].

As explained, patients with LCH masquerading HS mainly manifested skin ulceration and nodules [7,[13], [14], [15], [16], [17], [18], [19], [20], [21], [22], [23], [24], [25], [26], [27]]. The presented case also manifested enlarged purulent hidradenitis in the axilla and groin regions.

One patient had sexual dysfunction [16]. Polyuria, polydipsia, DI, and lung involvement were among frequent symptoms of these patients [7,[13], [14], [15], [16], [17], [18], [19], [20], [21], [22], [23], [24], [25], [26], [27]]. The presented case had a decline of libido, gynecomastia, moon face, polydipsia and polyuria.

The histopathological investigations of LCH reveal a high concentration of histiocytic mononuclear cells and multinucleated giant cells, extending from the dermis to the subcutaneous tissue [5,[11], [12], [13], [14], [15], [16], [17], [18], [19], [20], [21], [22], [23], [24], [25]]. Furthermore, the histiocytic cell populations tested positive for CD1a and S-100 proteins. The pathology results of our case were consistent with these findings.

Characteristic neuroimaging findings in individuals include involvement of the hypothalamic-pituitary area, thickening of the infundibulum, and the absence of a bright spot in the posterior pituitary. Additionally, neuroimaging may reveal enlargement and enhancement of the pineal gland, thickening and enhancement of the choroid plexus, as well as intraparenchymal masses [28].

The approach to treating LCH varies based on the extent and severity of the disease [29]. In case of more extensive involvement, systemic therapy is required [30]. Treatment for LCH CNS disease typically involves a standard LCH regimen, which is effective for tumorous lesions and new-onset diabetes insipidus. This regimen may include vinblastine and prednisone, or single-agent cladribine [31,32]. However, the presented case responded successfully to the surgical intervention without any complications or remission during the time of follow-ups.

Additionally, a case of a 19-year-old diagnosed with central DI that progressed to panhypopituitarism and presented with a Sellar-suprasellar mass, initially mistaken for inflammatory bowel disease (IBD), highlights the diverse and misleading symptoms associated with LCH. [5] Both cases emphasize hormonal imbalances, particularly in the hypothalamic-pituitary axis, as early indicators of Langerhans Cell Histiocytosis (LCH). LCH initially presented as hidradenitis suppurativa (HS) or inflammatory bowel disease (IBD), leading to intensive diagnostic efforts following the failure of standard treatments. The disparity between the skin-related symptoms in one case and the gastrointestinal symptoms in another underscores LCH's varied presentation. This variation highlights the importance for medical professionals to consider LCH when faced with atypical inflammatory symptoms resistant to conventional treatment.

This example illustrates the challenging diagnostic aspects of LCH, especially when it presents with symptoms similar to common skin conditions like hidradenitis suppurativa. The coming together of clinical features often leads to incorrect diagnoses and delayed treatment options. In this case, despite the patient's initial misdiagnosis, surgical intervention led to positive results in the end. This situation highlights the necessity for healthcare providers to remain vigilant when treating chronic skin conditions that do not respond to usual treatments, as they could mask the existence of underlying systemic diseases like LCH. A cooperative method involving dermatology, endocrinology, and pathology expertise is necessary for accurate diagnoses and timely treatment. As shown in this instance, early detection and tailored treatment methods, such as surgery, when necessary, can greatly improve patient outcomes, particularly in cases of multifocal or multisystemic LCH.

## 7 Conclusion

In conclusion, this case emphasizes the significance of acknowledging LCH in individuals exhibiting persistent dermatological manifestations, especially when conventional therapeutic modalities prove ineffective. The diagnostic intricacies associated with LCH, which frequently resemble more prevalent disorders such as hidradenitis suppurativa, necessitate an interdisciplinary methodology for precise diagnosis and subsequent treatment. This case illustrates the efficacy of surgical intervention while underscoring the imperative for increased awareness regarding the heterogeneous presentations of LCH. Timely identification and appropriate management are pivotal in enhancing patient prognoses, particularly for those exhibiting multifocal and multisystem involvement.

## Author contribution

Ibrahim Mohammadzadeh (IM), Narges Bazgir (NB), Behnaz Niroomand (BN), and Seyed Ali Mousavinejad (SAM) conceptualized the manuscript, reviewed the literature, and wrote the original draft. IM and BN designed the study. SAM and Nader Akbari and Ghaem Khodabakhsh performed the surgical procedure. IM and NB edited the manuscript. IM and SAM supervised the entire study process. All authors read and approved the final manuscript.

## Consent

The authors of this manuscript confirm that written informed consent has been obtained from the patient for the publication of the case report and any accompanying images. The consent process involved providing the patient with clear and understandable information about the study's purpose, procedures, potential risks, and benefits. The patient had the opportunity to ask questions and have their concerns addressed before voluntarily agreeing to participate in the publication of their case. A copy of the written consent form is available for review by the editor-in-chief of this journal upon request, ensuring transparency and accountability in the consent process.

## Ethical approval

Ethical approval is not required since our patient's treatment was based on approved options, and it was not found to be controversial, according to the Ethics Committee of our institution.

## Guarantor

Seyed Ali Mousavinejad.

## Funding

There is no funding source.

## Declaration of competing interest

Authors declared no personal or financial conflicts of interest.
